# Frequency of the rs2015 (T>G) and rs2241703 (G>A) polymorphisms in the miRNA-SIRT2 gene in type 2 diabetes mellitus in Saudi Arabia

**DOI:** 10.15537/smj.2023.44.4.20220863

**Published:** 2023-04

**Authors:** Nahla A. Alshaikh, Siddig I. Abdelwahab, Mahmoud M. Habibullah, Ahmed A. Jerah, Mohammed A. Bakri, Yahia A. Kaabi

**Affiliations:** *From Medical Laboratory Technology Department (Alshaikh, Habibullah, Jerah, Kaabi), Faculty of Applied Medical Sciences, Jazan University; from the Medical Research Center (Abdelwahab), Jazan University; and from the Department of Medical Laboratories (Bakri), King Fahd Central Hospital, Jizan, Kingdom of Saudi Arabia.*

**Keywords:** *SIRT2*, polymorphism, T2DM, Saudi Arabia

## Abstract

**Objectives:**

To investigate the prevalence of rs2015 (T>G) and rs2241703 (G>A) polymorphisms in the miRNA-SIRT2 gene in Saudi Arabia and their possible associations with type 2 diabetes mellitus (T2DM).

**Methods:**

Blood samples were collected from 428 participants from Jazan University Hospital, Jazan, Saudi Arabia between September 2021 and June 2022 and subjected to TaqMan single-nucleotide polymorphisms (SNP) genotyping assay for rs241703 (G>A) and rs2015 (G>T). Genotype frequencies were determined in control (n=217).

**Results:**

The A allele of rs2241703 was undetected in our population, and all samples carried the GG genotype. The rs2015 SNP frequency was 29.4% for GG, 45.6% for GT, and 24% for TT. However, logistic regression analysis of the dominant inheritance model showed no association between the T allele and T2DM calculated odds ratio [OR]=0.80, 95% confidence interval=0.53 to 1.20, *p*=0.301).

**Conclusion:**

Although rs2241703 SNP of Sirtuins 2 is not present, rs2015 SNP is highly prevalent in Saudi Arabia, but no direct link was identified with T2DM.

**T**ype 2 diabetes mellitus (T2DM) has a strikingly high incidence rate in Saudi Arabia posing a serious threat to the economy and public health.^1,2^ Type 2 diabetes mellitus is a chronic disease of carbohydrates metabolism characterized by a hyperglycemic state associated with acute and chronic complications.^3^ Dysregulation of metabolic pathways in T2DM is mainly attributed to insulin resistance.^4^ Increased oxidative stress, chronic inflammation and impaired mitochondrial function during aging are all linked to the pathogenesis of T2DM and age-related insulin resistance.^5-7^

Sirtuins (SIRTs) are a conserved family of cellular enzymes known as; histone deacetylases that play a crucial role in regulating genetic information.^8^ In mammals, 7 (SIRT1 to SIRT7) are involved in a broad range of biological activities and promote longevity.^9^ *Sirtuin 2* expressed primarily in the cytoplasm, but it can also be found in the mitochondria and nuclei.^10^ Although *SIRT2* is the least studied SIRT protein, its role in numerous physiological and pathological conditions is well-reported.^10,11^ Experiments in rats showed that impaired pancreatic insulin release may result from genetic deletion or inhibition of *SIRT2*.^12^ Additionally, *SIRT2* knockout in mice contributed to insulin resistance, mitochondrial depletion, and impaired redox balance.^13^ Other studies also suggest that *SIRT2* regulates gluconeogenesis and insulin-stimulated glucose uptake.^14,15^

Two common single-nucleotide polymorphisms (SNPs) reported in the 3′ untranslated region (UTR) of *SIRT2* are substitution mutation rs2241703 (G>A) and transversion substitution mutation rs2015 (G>T) or (C>A). These SNPs can affect the binding ability of microRNAs to *SIRT2* mRNA, thereby downregulating *SIRT2* protein expression and contributing to disease development.^16,17^ Previous studies have linked rs2015 and rs2241703 in the 3′ UTR of *SIRT2* to Alzheimer’s disease and Parkinson’s disease 16-18. However, the association between *SIRT2* rs2015 (G>T) and rs2241703 (C>A) and T2DM susceptibility has not been studied. Therefore, in this study, we aimed to estimate the prevalence of these SNPs in Saudi Arabian population and determine any possible correlation with the risk of developing T2DM.

## Methods

In this case-control study, a total of 428 volunteers were participated, comprising 217 healthy controls and 211 T2DM patients. They were recruited from Jazan University Hospital, Jazan, Saudi Arabia between September 2021 and June 2022. The selection criterion was: Saudi Arabian citizens aged ≥ 35 years with or without T2DM. Physically challenged individuals, pregnant women, and extremely obese people were excluded from the study. The study protocol was revised and approved by the Ethics Committee of Jazan University (REC-43/02/017) according to principles of Helsinki Declaration. Written informed consent was obtained from all participants before the sampling. The following demographic data and basic clinical characteristics were collected for each volunteer: gender, age, weight, height, and family history of T2DM at the time of sample collection. Body mass index (BMI) was obtained mathematically according to the formula: BMI=weight (kg)/height (m^2^). The level of fasting plasma glucose (FPG) was measured using a colorimetric method-based glucose oxidase. The percentage of glycated hemoglobin (HbA1c) was determined using a quantitative turbidimetric inhibition immunoassay.

### Sample preparation

Venous blood was obtained from all participants in 5 mL Becton Dickinson (BD) ethylenediaminetetraacetic acid vacutainer tubes. Genomic DNA (gDNA) was isolated from white blood cells found in 200 µL of whole-blood sample using the GeneJET gDNA isolation kit (Thermo Scientific, Waltham, MA, USA). The amount of DNA extracted was determined using a Thermo Scientific NanoDrop One microvolume ultraviolet-visible spectrophotometer at medical research center at Jazan University. The DNA concentration ranged from 20 to 30 ng/µL in all samples. The quality of the extracted DNA was determined by measuring (A_260_/A_280_) ratios and running on one percent agarose gel electrophoresis. If not used directly, the DNA samples were then stored frozen at -20°C until subsequent genetic testing.

### Sirtuin2 SNP analyses

Sirtuin2 rs2241703 and rs2015 genotyping was performed in 96-well plates using TaqMan allelic discrimination assays; assay ID: C__15873898_10 and C____104093_1_, respectively (Thermo Fisher Applied Biosystems, Foster City, CA, USA). Real-time polymerase chain reaction (RT-PCR) using specific TaqMan probes was carried out using the ABI 7300 real-time PCR thermal cycler (Applied Biosystems). The PCR was carried out in a total reaction mixture volume of 25 µL, comprising 15 ng gDNA diluted in 11.25 µL nuclease-free water and 12.5 µL 2× TaqMan™ genotyping master mix premixed with 1.25 µL 20× assay working stock of the TaqMan SNP genotyping assay reagent.

The assay reagent contains specific primers and allele-specific probes labeled with VIC (for the rs2241703 A or rs2015 G alleles) and FAM (for the rs2241703 G or rs2015 T alleles) fluorescent dyes. The DNA for the SNP detection was amplified according to the following protocol: step 1 (Pre-PCR read); 30-seconds reading of the baseline fluorescence at 60°C; step 2, (PCR amplification); one cycle of 10-minutes DNA denaturation at 95°C, followed by 40 cycles of 15-seconds DNA denaturation at 92°C and 1-minute extension at 60°C; and step 3, (Post-PCR read); 30-seconds reading of final fluorescence at 60°C.

### Statistical analysis

We performed all statistical analyses using GraphPad Prism 9 software (San Diego, CA, USA). For continuous values, data were presented as means ± standard deviation (SD) and for categorical values are presented as absolute numbers with their percentages. Gender distribution and the presence of family history are presented as numerical values and frequencies and were compared using the Chi-square (χ2) test. Continuous data (age, weight, height, BMI, and FPG and HbA1c levels) are expressed as means ± SD and the student’s t-test was used to compare between means.

Hardy–Weinberg Equilibrium (HWE) test was used to compare the observed and expected frequencies of *SIRT2* rs2015 (G>T) and rs2241703 (G>A) SNPs in both the control and T2DM group using a χ2 test. Additionally, binary logistic regression analysis was used to test possible relationship between these SNPs and T2DM risk at different inheritance models: codominant, dominant, recessive, and additive, by calculating odds ratios (ORs) at a 95% confidence interval (CI). All tests were 2-tailed, and a *p*-value of ≤0.05 considered statistically significance.

## Results

We performed genotyping for *SIRT2* rs2241703 (G>A) and rs2015 (G>T) in 428 participants from Saudi Arabia. All extracted DNA samples used showed an A260/A280 ratio >1.8, and the stained agarose gel images showed intact DNA bands in both the healthy control (n=217) and T2DM (n=211) groups. The base line characteristics of the total study population and of each group are presented in Table 1. The study included 198 male (46.3%) and 230 female (53.7%) participants. Gender distribution between the control and T2DM patients was similar, and the χ2 test showed no significant difference (*p*=0.905). Except for height, all other demographic and biochemical parameters showed significant differences between the healthy control and T2DM groups (Table 1).

**Table 1:** - Characteristics of study participants.

Variables	Total samples (n=428, 100%)	Control group (n=217, 50.7%)	T2DM group (n=211, 49.3%)	*P*-value
***Gender, n (%)***				
Male	198 (46.3)	101 (46.5)	97 (46.0)	0.905*
Female	230 (53.7)	116 (53.5)	114 (54.0)	
Age (years)	53.5±13.3	50.1±13.6	57.1±11.9	<0.0001
Weight (kg)	72.6±13.4	70.3±13.1	75.1±13.4	0.0005
Height (cm)	161±8.04	162±7.06	161±8.86	0.1333
BMI (kg/m2)	27.6±5.05	26.5±5.07	28.8±4.77	<0.0001
FPG (mmol/L)	7.43±2.19	5.47±0.546	8.97±1.71	<0.0001
HbA1c (%)	9.44±5.50	5.65±1.04	12.5±5.73	<0.0001
Family history, n (%)	119 (27.8)	38 (17.5)	81 (38.3)	<0.0001*

Data represent means±standard deviation unless otherwise specified. T2DM: type 2 diabetes mellitus, BMI: body mass index, FPG: fasting plasma glucose, HbA1c: glycated hemoglobin. *Chi-square (χ2) test.

In this study, we were unable to detect the A allele of rs2241703 (G>A) in any of the tested samples; therefore, all samples had the homozygous GG genotype. However, all variants of *SIRT2* rs2015 (G>T) were detected in our population. In the total study population, the frequency of the rs2015 variants was 29.4% for the homozygous GG, 45.6% for the heterozygous GT, and 24% for the homozygous TT genotypes (Table 2). The Hardy–Weinberg equilibrium (*p*=0.172) showed that the genotype distribution in the healthy control group was 27.2% for GG, 47% for GT, and 25.8% for TT.

**Table 2:** - Genotype and allele distribution of sirtuin2 (*SIRT2*) rs2015 and rs2241703 in control and type 2 diabetes mellitus (T2DM) groups.

Genotype/Allele	Total N=428	Control n=217	T2DM n=211	HWE *P*-value	*P*-value*
rs2015					
GG	126 (29.4)	59 (27.2)	67 (31.7)	0.172	0.308
GT	195 (45.6)	102 (47.0)	93 (44.1)		0.548
TT	107 (25.0)	56 (25.8)	51 (24.2)		0.703
G	447 (52.2)	220 (50.7)	227 (53.8)		0.521
T	409 (47.8)	214 (49.3)	195 (46.2)		0.521
rs2241703					
GG	428 (100)	217 (100)	211 (100)	NA	NA
GA	0	0	0		NA
AA	0	0	0		NA
G	856 (100)	434 (100)	411 (100)		NA
A	0	0	0		NA

Values are presented as number and percentages (%). *Chi-square χ2) test. NA: not applicable

A similar distribution was also found in the T2DM group (GG, 31.7%; GT, 44.1%, and TT, 24.2%; Table 2). The proportions test showed no significant difference between the genotype distribution in both study groups (all *p*>0.300). The G allele frequency was 50.7% in the control group and 53.8% in the T2DM group. The T allele frequency was 49.3% in the control and 46.2% in the T2DM group, and no significant difference was observed (*p*=0.521) between the 2 groups. Binomial logistic regression analysis was applied on *SIRT2* rs2015 in the T2DM and control groups in different inheritance models to further examine possible associations between the rs2015 (G>T) variants and T2DM (Table 3).

**Table 3:** - Binomial logistic regression analysis of type 2 diabetes mellitus (T2DM) and control groups

Model	Genotype/allele	OR (95% CI)	*P*-value
Codominant	GT vs. GG	0.803 (0.51 to 1.25)	0.362
	TT vs. GG	0.802 (0.47 to 1.33)	0.402
Dominant	GT+TT vs. GG	0.803 (0.53 to 1.20)	0.301
Recessive	TT vs. GT+GG	1.091 (0.71 to 1.67)	0.696
Additive	T	0.883 (0.67 to 1.16)	0.374

OR: odd ratio, CI: confidence interval, vs: versus

In the codominant inheritance model of GT versus (vs.) GG, the calculated OR was 0.80, with a 95% CI of 0.51 to 1.25 (*p*=0.362). In addition, for the TT vs. GG model, OR=0.80 (95% CI: 0.47 to 1.33, *p*=0.402), and none of the results were significant. In the dominant inheritance model for GT+TT vs. GG, the calculated OR was 0.80 (95% CI: 0.53 to 1.20, *p*=0.301), which was not significant. In the recessive model of inheritance for TT vs. GT+GG, the OR was 1.091 (95% CI: 0.71 to 1.67, *p*=0.696), which was also not significant. The allelic distribution was similar in the additive inheritance models (Table 3).

## Discussion

This study estimated the prevalence of *SIRT2* rs2241703 (G>A) and rs2015 (G>T) SNPs in a Saudi Arabian population and tested the associated risk of developing T2DM. However, we failed to detect the A allele of rs2241703 (G>A) in the total study population and all samples carried the homozygous GG genotype. This observation indicates that this SNP is extremely rare or absent in the Saudi Arabian population. The SNPedia database also reports the absence of this A allele in Europeans and Africans; however, the A allele has the highest frequencies in Asian populations such as the Japanese (41.9%) and Han Chinese (15.6%). In the rs2015 (G>T) SNP, the frequency of the T allele in our population was 47.8%, which is close to that found in Japanese (53%) and Chinese (55%) populations where this SNP is predominant.^16,17^

The case control design of this study also allowed us to determine any potential relationship between rs2015 (G>T) and T2DM. The results of the binomial logistic regression analysis of different patterns of inheritance (codominant, dominant, recessive, and additive) demonstrated a lack of association between the T allele of the *SIRT2* promoter rs2015 (G>T) polymorphism and T2DM in the study population. A previous study based on Chinese population reported a direct association of rs2015 (G>T) polymorphism and the risk of Parkinson’s disease.^17^ Furthermore, polymorphism of *SIRT2* rs2015 is associated with Alzheimer’s disease.^16^ Along with *SIRT2* rs2241703 and rs2015 polymorphisms, 3 other SNPs were found to upregulate the transcriptional activity of *SIRT2* that may contribute to human disease.^19^ Moreover, some polymorphisms found on the *SIRT2* promoter region, namely; P-MU1, P-MU2, and P-MU3, may interfere with transcription factor binding and increase susceptibility to T2DM.^20^ These studies provide considerable evidence supporting the association between *SIRT2* polymorphism and T2DM risk. Furthermore, this observation emphasizes the need for more research investigating the frequency and association of other *SIRT2* SNPs and T2DM in the Saudi population because the present study focused only on 2 SNPs, rs2015 and rs2241703.

### Study limitation

Despite the limited number of volunteers participated in the study, our early findings will need to be confirmed with large-scale investigations using novel methodologies, other than the standard case-control study design. The significant difference in age group between T2DM and control groups can be also a limitation of this study. This could be related to the fact that most of T2DM patients visiting the clinic were in their fifties or sixties. Additionally, the study exhibited higher number of females than males in total study population, however, the distribution of gender frequency was similar between the 2 study groups.

In conclusion, the current study reported the prevalence of the *SIRT2* rs2241703 (G>A) and rs2015 (G>T) polymorphisms in the Saudi Arabian population. We did not detect the minor A allele of rs2241703 in our study population and the homozygous GG genotype found to be the most prevalent genotype. Therefore, we ruled out the association between this SNP and T2DM. In contrast, we identified a high prevalence of the T allele of rs2015, similar to that in Asian populations. Although we did not identify a direct link between rs2015 (G>T) and the likelihood of developing T2DM, more studies are required to explore the association between rs2015 and other chronic age-related disorders, especially neurodegenerative diseases.
